# Editorial: Machine Learning and Wearable Technology in Sleep Medicine

**DOI:** 10.3389/fdgth.2022.845879

**Published:** 2022-03-02

**Authors:** Timo Leppänen, Carolina Varon, Massimiliano de Zambotti, Sami Myllymaa

**Affiliations:** ^1^Department of Applied Physics, University of Eastern Finland, Kuopio, Finland; ^2^Diagnostic Imaging Center, Kuopio University Hospital, Kuopio, Finland; ^3^School of Information Technology and Electrical Engineering, The University of Queensland, Brisbane, QLD, Australia; ^4^Microgravity Research Center, Service Chimie-Physique, Université Libre De Bruxelles, Brussels, Belgium; ^5^SRI International, Menlo Park, CA, United States

**Keywords:** artificial intelligence, machine learning, wearable sensors, sleep medicine, sleep disorders, deep learning, digital health

The “*Machine Learning and Wearable Technology in Sleep Medicine*” Research Topic consists of research articles related to the utilization of artificial intelligence (AI) to analyze sleep recordings, estimate sleep quality, and identify sleep disorders-related adverse health consequences as well as articles related to wearable sleep sensors and systems. This Editorial summarizes some key points of the Research Topic.

Short and interrupted sleep is related to several health-threatening medical conditions. Nearly one billion people worldwide suffer from obstructive sleep apnea (OSA) that causes a major burden to the affected individuals but also to the society and economy (1). However, most of the affected individuals remain undiagnosed mainly due to the unawareness of the disease and lack of readily available screening tools (1). This is, the current diagnostic methods in clinical sleep medicine are laborious, expensive, and too complex for self-application, thus not optimally suited for diagnosing a vastly increasing number of patients.

Polysomnography (PSG) is the gold standard method for the diagnosis and severity estimation of various sleep disorders. Multiple signals are recorded in PSG and thus, it contains a vast amount of information on human physiology. However, in many cases, only a limited amount of information is used for clinical decision-making. Also, PSGs are usually manually annotated making analysis of PSGs time-consuming, laborious, and expensive. To alleviate the manual workload, automated analysis methods have been the subject of extensive research in recent years. In this Research Topic, Van der Plas et al. introduced an automatic sleep staging model that utilizes pre-defined in-lab PSG signal features, mainly those defined by the American Academy of Sleep Medicine. The overall accuracy of their model was good (82.7%) and it is very well on par with the inter-rater agreement between two expert scorers.

The high number of electrodes and sensors used during a PSG are known to disrupt patients' sleep, making its use at home cumbersome (2). Thus, to make PSGs more convenient and less disruptive, automated analysis tools have been developed for the reduced number of signals and consumer-grade sleep trackers. Huysmans et al. developed a deep learning-based model for sleep-wake classification utilizing only cardiac and respiratory signals. Their model achieved a moderate sleep-wake classification accuracy [Cohen's Kappa (κ) of 0.48–0.51] depending on OSA severity. Also, Liang and Alberto Chapa-Martell developed a two-level classifier to identify sleep stages (light sleep, deep sleep, rapid eye movement sleep, and wake) from data available from consumer-grade activity trackers (i.e., steps, heart rate, and sleep metrics). As also their model achieved moderate overall epoch-wise accuracy of 0.731% ± 0.119% (κ = 0.433 ± 0.212), it is evident that some challenges are still to be overcome when utilizing this type of data in AI-based sleep staging models. However, novel automatic sleep staging methods utilizing the reduced amount of information compared to conventional PSG are highly promising, and existing efforts provide a good basis for further development of such kinds of automatic analysis tools.

As said, PSG is a bit too heavy measurement for the diagnosis of several sleep disorders, such as OSA. Therefore, in many countries, the home sleep apnea test (HSAT) is used instead. Basically, HSAT is the same measurement as PSG without recording of electroencephalogram (EEG), electrooculogram (EOG), chin electromyogram (EMG), and electrocardiogram (ECG). This makes the HSAT measurement setup lighter, thus more feasible and readily available. However, as a downside, the lack of EEG prevents the accurate determination of sleep stages and can affect the diagnosis or severity estimation of OSA (3). To overcome this, several researchers have been developing self-applicable EEG measurement sensors and devices to be used alongside HSAT. One such solution is a flex-printed ear-EEG sensor developed by da Silva Souto et al., from which the data is wirelessly collected with a smartphone. This solution was found to have a substantial agreement (κ = 0.67) in hypnograms with PSG-based sleep staging (Fpz and EOG). This suggests that sleep stages can be manually identified from the signals collected with a simple, self-applicable system around the ear and justifies the further development of automatic tools utilizing such data.

In contrast to HSAT, even lighter measurement devices exist to monitor sleep and circadian rhythms. Actigraphy devices are movement-based devices able to estimate sleep and wake periods and as stated in the review by Lujan et al., such devices are readily available for the general population. In addition, a new generation of actigraphy devices has even more potential in clinical practice, as the recent developments of the devices to include new sensors have enabled measurements of heart rate (HR) and heart rate variability. As found by Huang et al., both HR and activity can be used to estimate circadian rhythms but while used together, the estimate is more reliable.

One future direction in sleep medicine could be the more widespread utilization of pulse oximeter data or voice analysis. For example, photoplethysmography (PPG) signal formation is based on the changes in blood volume in peripheral arteries measured from the skin surface, e.g., from a fingertip (4). The PPG signal carries information on breathing, brain activity, and heart functioning and thus, PPG could be used as a surrogate for several physiological phenomena. Furthermore, PPG could also provide valuable information on the risk of OSA-related co-morbidities. For example, Ji et al. developed a promising machine learning-based approach, utilizing only PPG signal features, to predict vaso-occlusive crises in patients with sickle cell disease. In addition, previous studies have shown promising results related to voice-based estimation on chronic and immediate sleepiness, however, as highlighted by Martin et al. these applications are not ready yet for clinical use.

In summary, the latest advances in wearable sensor technology, digital signal processing, and AI can provide great opportunities to increase the availability of sleep recordings, simplify the measurement protocol, and enhance the accuracy of clinical decision-making. After accurate and critical validation and regulatory processes, new methods can be used to monitor sleep quality, detect and estimate the severity of various sleep disorders, and better identify those individuals with the greatest need for treatment, i.e., high risk for adverse health consequences.

## Author Contributions

TL wrote the first draft of the Editorial. CV, MZ, and SM contributed to the writing of the Editorial. All authors have reviewed the manuscript critically and accepted the latest version to be submitted.

## Conflict of Interest

TL has received research funding from the European Union's Horizon 2020 Research and Innovation Programme (grant 965417), the Academy of Finland (project 323536), Nordforsk (NordSleep project 90458) *via* Business Finland (5133/31/2018), and the Research Committee of the Kuopio University Hospital Catchment Area for the State Research Funding (projects 5041767 and 5041794). CV has received research funding from the European Space Agency, Belspo (Prodex-NEPTUNE). MZ was employed by SRI International, has received research funding unrelated to this work from Noctrix Health, Inc. and Lisa Health Inc., is a co-founder of Lisa Health Inc., and has ownership of shares in Lisa Health. SM has received research funding from Nordforsk (NordSleep project 90458) *via* Business Finland (5133/31/2018) and the Research Committee of the Kuopio University Hospital Catchment Area for the State Research Funding (project 5041797).

## Publisher's Note

All claims expressed in this article are solely those of the authors and do not necessarily represent those of their affiliated organizations, or those of the publisher, the editors and the reviewers. Any product that may be evaluated in this article, or claim that may be made by its manufacturer, is not guaranteed or endorsed by the publisher.
